# Supplementary material for: BCDnet: Parallel heterogeneous eight-class classification model of breast pathology
Source: PLoS One. 2021 Jul 12;16(7):e0253764. doi: 10.1371/journal.pone.0253764 (PMC8274904; doi:10.1371/journal.pone.0253764)
Supplement: Attachment — Submitted filename: PONE-D-20-37399 REVIEW COMMENTS.docx [file pone.0253764.s001.docx]

1) The overall grammar and sentence framing in the paper are very weak, making the article quite unreadable. Grammar checks were not done at all.
2) The author should try to give data of the study region too, in the Introduction.
3) Example:

line 83… "Most of the research focuses on 84 relatively simple two classification"…. Here it appears that the author's research focuses on this.

Line 85… "…moving from two classifications….." maybe binary is a better term

Line 87…. "Improving the accuracy of the model by improving the data set has also been paid more and more attention". How has the data set been improved? Is this sentence part of literature, or this work?... is unclear.

Line 165… "If you load the weights and freeze all the convolutional layer weights, because the loaded weights come from the training of the ImageNet image set, the samples in the ImageNet image set have little correlation with the samples in the pathological tissue image set, so the experimental results of strategy 3 are poor." Very bad sentence framing.

Line 302… "…sample number distribution…." What does this mean?

4) Line 103…"….using random non-repetitive segmentation of the original image sample to enhance the data for the small sample can significantly improve the model performance". This sentence must have the support of citation.
5) Line 141… "Choose pre-trained models" Title should be appropriately named. Maybe Choice of pre-trained models.
6) It is not understood how Figure 2 describes the model structure. Further, no description is added of the labels in the figure.
7) Table 2 mentions the raw dataset numbers. There is no breakup of what the enhanced dataset numbers are.
8) Line 295… "…The abscissa is the xx patien…” What is this xx?
9) The paper has commas and blanks spaced out at will. Seems like a very hurriedly written paper.
10) The paper's only novelty is the 8-class classification, which is interesting, and hence carries a chance for re-submission.
11) But, if a new AI method/theory is being reported in the paper, it should be compared and validated against at least one other common data set (bench mark datasets) for which a published study exists using at least one other method/approach, and preferably a method/approach which has been widely used in the field.
12) Also, baselines are not strong (e.g., did not compare with state-of-the-art approaches)
