# Supplementary material for: BCDnet: Parallel heterogeneous eight-class classification model of breast pathology
Source: PLoS One. 2021 Jul 12;16(7):e0253764. doi: 10.1371/journal.pone.0253764 (PMC8274904; doi:10.1371/journal.pone.0253764)
Supplement: Attachment — Submitted filename: Response to Reviewers.docx [file pone.0253764.s002.docx]

**Dear editor and two reviewers:**

First of all, thanks for your comments and suggestions on this article, and sincere thanks for your guidance and help. The two reviewers showed a high level of professionalism and reviewed this article very carefully. The comments and suggestions given by the reviewers are very pertinent., We have learned a lot from these opinions. In response to your comments and suggestions, we have carefully revised the article repeatedly.

Our Manuscript Number: PONE-D-20-37399

Title: BCDnet: Parallel Heterogeneous Eight-category Automatic Diagnosis Model of Breast Pathology

New Title: BCDnet: Parallel heterogeneous eight-class classification model of breast pathology

Here is our reply:

With reference to the comments of the two reviewers, we slightly revised the title of the original manuscript in order to maintain the same terminology in the full text.

To Editor: Regarding the submitted figures 4 and 6 contain copyrighted images, these two figures have been deleted in the revised manuscript.

To reviewers: Regarding the comments made by the two reviewers that the English language of this article is bad. The author agrees with the reviewer's opinion, and the revised manuscript has been revised repeatedly, trying to clarify the meaning to be expressed. Here, I sincerely apologize for the trouble caused by the original article to the two reviewers.

### To Reviewer 1：

line 83… "Most of the research focuses on relatively simple two classification"…. Here it appears that the author's research focuses on this.

**Reply**: This sentence does not express the meaning clearly in this article. The original intention is that most of the published research results focus on the binary classification of the pathological tissues of breast cancer. The revised draft has been revised.

Line 85… "…moving from two classifications….." maybe binary is a better term，

**Reply:** The reviewer’s opinion is correct. It is more accurate to replace two classifications with binary classification. The revised draft has been revised.

"Improving the accuracy of the model by improving the data set has also been paid more and more attention". How has the data set been improved? Is this sentence part of literature, or this work?... is unclear.

**Reply:** There are generally two ways to improve the data set: (i) Some samples in the data set have errors and need to do some processing on the used data set, such as checking data consistency, dealing with invalid values and missing values, etc. (ii) In some data Set, the distribution of the number of classified samples is extremely uneven. A data set with an unbalanced sample size may affect the effect of machine learning, and some measures need to be taken, such as adopting some methods to increase the data with a small number of samples, so that the number of samples for classification is evenly distributed. In response to this opinion, the revised draft has been revised in terms of expression.

Line 165… "If you load the weights and freeze all the convolutional layer weights, because the loaded weights come from the training of the ImageNet image set, the samples in the ImageNet image set have little correlation with the samples in the pathological tissue image set, so the experimental results of strategy 3 are poor." Very bad sentence framing.

**Reply:** The sentence in the original text is too long and has been revised in the revised version.
Line 302… "…sample number distribution…." What does this mean? Reply: This sentence is not clearly expressed in this article. I originally wanted to express: "The ratio of the total number of samples of benign tumor pathological tissue to the total number of samples of malignant tumor pathological tissue is 1:2". This expression has been revised in the revised draft.

Line 103…"….using random non-repetitive segmentation of the original image sample to enhance the data for the small sample can significantly improve the model performance". This sentence must have the support of citation.

**Reply:** The previous sentence of this sentence is not clearly expressed in this article. What the author wants to express is: This is one of the conclusions drawn from the results of multiple experiments in this article. The revised draft has been rephrased appropriately.

Line 141… "Choose pre-trained models" Title should be appropriately named. Maybe Choice of pre-trained models.

**Reply:** It is more appropriate to use Choice of pre-trained models as the title, and the revised version has been revised.

It is not understood how Figure 2 describes the model structure. Further, no description is added of the labels in the figure.

**Reply:** The original introduction is unorganized. This content and more have been added to the revised draft to fully introduce the model structure.

Table 2 mentions the raw dataset numbers. There is no breakup of what the enhanced dataset numbers are.

**Reply:** The original text is only a text description of the data enhancement, and the expression is not clear. In addition to modifying the text description, the revised draft also adds two tables. One table describes the number of samples in each category after the expanded data set, and the other describes the number of samples in each category after the balanced data set.

Line 295… "…The abscissa is the xx patien…” What is this xx?

**Reply:** This sentence is an explanation of the original Figure 5 (Figure 1 in the revised draft). Here xx refers to one of the 38 DC patients. Considering that the figure has been clearly expressed, this sentence is deleted in the revised draft.

The paper has commas and blanks spaced out at will. Seems like a very hurriedly written paper.

**Reply:** The reviewer is right. Some parts of this article are described unclearly, and even some parts are ambiguous. We apologize for causing reading difficulties to the reviewers. The revised manuscript strives to improve language expression in this regard.

The paper's only novelty is the 8-class classification, which is interesting, and hence carries a chance for re-submission.

Reply: We would like to express my sincere thanks to the reviewer for the opportunity to revise.

But, if a new AI method/theory is being reported in the paper, it should be compared and validated against at least one other common data set (bench mark datasets) for which a published study exists using at least one other method/approach, and preferably a method/approach which has been widely used in the field.

**Reply:** The suggestions given by the reviewers are good. Now use two classic classification models resnet50 and VGG16, and use the original model and the pre-trained model + fine-tuning two modes, repeat the experiment process of this article. The results of the two are compared with the model proposed in this paper. The revised draft has supplemented this content.

Also, baselines are not strong (e.g., did not compare with state-of-the-art approaches)，

**Reply:** Currently, there are few eight-class classification literatures on breast cancer pathological tissues, most of which are two-class classification and four-class classification. Therefore, the revised draft adds BCDnet and compares the best results of two classic pre-trained models + fine-tuning.

### To Reviewer 2:

Generally, research articles are structured with abstract, introduction, methods, results, discussions, and conclusion headings. Can the authors reorganize their manuscript according to these conventions?

**Reply:** The reviewer's opinion is very good. The full text of the revised manuscript has been re-arranged according to the reviewers’ comments.

Though some ideas presented by the authors can be understood, some paragraphs are difficult to understand. For example, this reviewer finds it difficult to understand what is being said on lines 101-109. There are also numerous instances of sentence fragments, run-on sentences, comma splices, etc. Please have someone who is fluent in English to proof read the manuscript.
**Reply:** There are grammatical errors in some sentences in the original text, and the expression is unclear, which caused reading difficulties to the reviewers. We apologize. The revised manuscript pays special attention to this. The full text of the revised manuscript has been revised many times in terms of language. We strive to express the content clearly and achieve correct grammar.

It is not clear in the "Choose pre-trained models" subsection how models are trained nor what results are reported in Table 1. The authors state that they are using strategies 2, 3, and 4 to pre-train on a small set of pathological tissues. What performance metric is being measured? Accuracy? How are large tissue images being processed by the fixed-input CNNs? Can the authors describe the distribution of the dataset before presenting results?

**Reply:** The reviewer's opinion is very good. The overall layout of the original text was unreasonable, and it was re-described in the revised manuscript, and its paragraph position in the article has also changed in the revised manuscript.

The authors do well describing Resnet50 and VGG16. This reviewer can understand that the 30th and 10th layers respectively are frozen as well as preceding layers. Then, deep layers are retrained. However, it is not clear what parameters are utilized to fine-tune these networks (learning rate, optimizer, weight biases, etc.). Furthermore, it is not clear what is the 30th layer or 10th layer of these networks. Generally when describing CNN layers, we prefer to refer to abstracted components of the network. For example, Resnet50 has five residual "blocks." It would help to clarify these sections.

**Reply:** The reviewer's opinions are very professional. In response to the reviewers' comments, we have uploaded the source code used in this article to github. Readers can view the parameter configuration and specific implementation in detail. The revised version adds the source code download address here. The description and explanation of the frozen layer have been revised in the revised manuscript according to the comments of the reviewer.

The resampling strategy proposed by the authors to combat class imbalances is well-founded. However, the description provided is not clear enough to reproduce the resampling strategy. Can the authors rephrase their description?

**Reply:** The revised draft adds two tables (Table 2, Table 3)to this, and also revised the specific instructions to make it clear.

Table 2 The distribution of various samples after the image segmentation method is used to expand the data set.

Table 3 The distribution of various samples after the data set is balanced.

The authors make it a point that class imbalance will be an issue, but given the results, it seems as if it hardly makes a difference. Yes, the majority of performance metrics are higher with augmentation but only a little bit. Can the authors provide some discussion on this?

**Reply:** The revised manuscript adds a discussion of the difference in precision of each classification of balanced data and unbalanced data, and analyzes the reasons for the difference in model accuracy based on balanced data and based on unbalanced data.

Minor
It is a bit unusual to compare lung and breast cancer as a race to first place on lines 40-41 on page 2. Please rephrase.

**Repl y:** "Lung cancer" has been deleted in the revised draft and the relevant expression has been renewed.

The transition from pathological diagnosis to image classification on lines 46-47 on page 2 is non-sequitur. Please rephrase.

**Reply:** The revised manuscript made changes to this paragraph to make it logical and overly natural.

What does "pre-screened" mean on line 233 page 7?

**Reply:** The original wording is inaccurate, and this paragraph has been revised in the revised draft.

What is meant by "mark" in Table 3 (page 10)?

**Reply:** The original wording is not exact, it should be "Label", the revised version has been revised here.

What is verification accuracy and loss?

**Reply:** The wording is inaccurate, it should be "validation accuracy and loss", the revised version here has been revised.

Can the authors represent Table 4 as a grouped bar graph? It is difficult to see the differences in performance with such a large table.

**Reply:** The reviewer's suggestion is very good, and the revised manuscript has been revised according to this suggestion. See Fig 6 of the revised manuscript. **Fig 6 Experimental results based on unbalanced data set and balanced data set.**

One of the columns for Dataset400X in Table 4 contain results rather than marks. Please fix.
**Reply:** This table has been replaced by Figure 6 in the revised draft. See Fig 6 of the revised manuscript.

Does Table 4 report cross-validation metrics?

**Reply: yes.**

We look forward to hearing from you regarding our submission. We would be glad to respond to any further questions and comments that you may have.

Best regards!

He Qingfang Cheng Guang Ju Huimin

2021-04-07
