# Supplementary material for: BCDnet: Parallel heterogeneous eight-class classification model of breast pathology
Source: PLoS One. 2021 Jul 12;16(7):e0253764. doi: 10.1371/journal.pone.0253764 (PMC8274904; doi:10.1371/journal.pone.0253764)
Supplement: Attachment — Submitted filename: Response to Reviewers.docx [file pone.0253764.s003.docx]

**Dear editor and reviewers:**

First of all, thanks for your comments and suggestions on this article, and sincere thanks for your guidance and help.

Two reviewers (Reviewer #2 and Reviewer #4) showed a high level of professionalism and gave positive opinions on the revised manuscript of this article.

Reviewer 3# gave many negative opinions, for which we make the following response.

“ 2. Is the manuscript technically sound, and do the data support the conclusions?

Reviewer #3: No”

**Reply**: Based on deep convolutional neural network, model fusion, and feature fusion technology, this paper proposes an eight-class classification of breast pathological tissue diagnosis model (BCDnet). Experiments were carried out on the public BreaKHis scientific research data set, and the experimental results proved that BCDnet achieved eight-class classification of breast cancer pathological tissues, and the classification accuracy was higher than the published research results.

“3. Has the statistical analysis been performed appropriately and rigorously?

Reviewer #3: No”

**Reply:** The article provides detailed data records, data grouping records and experimental results analysis. It also provides a comparative analysis of other models and the BCDnet model. Such as Table 1 Overview of BreaKHis data set, Table 2 The distribution of various samples after the image segmentation method is used to expand the data set, Table 3 The distribution of various samples after the data set is balanced, Fig 5. Recording curve of training process based on 100X, 100X+ data set, Fig 6 Experimental results based on unbalanced data set and balanced data set, Fig 7. Experimental results of eight-class classification precision based on 40X and 40X+ data sets, Fig 8. Comparison of accuracy of R50pt, V16pt and BCD based on unbalanced data sets and balanced data sets and Fig 9. Precision curve of eight classifications based on 40X and 40X+ , etc.

“5. Is the manuscript presented in an intelligible fashion and written in standard English?

Reviewer #3: No”

**Reply:** We do have shortcomings in English, and we need to improve our language skills. We had repeatedly revised the original revised manuscript, trying to express the content clearly. In this revised manuscript, we have looked for errors in language expression and revised them, and we also invited English professionals to review them to further improve our English expression skills.

“Reviewer #3: This is the review of the paper titled " BCDnet: Parallel heterogeneous eight-class classification model of breast pathology"
The paper has many flaws which I will list some of them.
1- poor novelty, there is nothing new in the paper.
2- poor presentation, many sentences are ambiguous.
3- the used dataset is old and very high results achieved on the dataset.
4- no confusion matrics are presented and visualization of the learned filters.
5- Poor English”

**About** 1- poor novelty, there is nothing new in the paper.

**Reply:** We use advanced technology in deep learning (model fusion, etc.) to independently design BCDnet to solve the problem of low multi-class recognition rate of breast cancer pathological tissues. Judging from the results published so far, our model and method are leading.

**About** 2- poor presentation, many sentences are ambiguous.

**Reply:** The original revised manuscript has been repeatedly revised. In this revision, the language has continued to be improved.
**About** 3- the used dataset is old and very high results achieved on the dataset.

**Reply:** Since 2015, Spanhol et al. published the BreaKHis breast cancer pathology image data set [10]. Based on this data set, a series of research results have been achieved in breast cancer recognition using convolutional neural networks. The data set used in this article (BreaKHis data set) is a public scientific research data set, and now it is widely used to train and test breast cancer diagnostic models.

Our model adopts the current popular model fusion and other technologies, and adopts improved experimental methods according to the characteristics of pathological tissues. Therefore, the experimental results reach a good level. The experimental model, operating procedure and data set of this article have been published, and we hope that everyone will evaluate to further improve the experimental results.

**About**  4- no confusion matrics are presented and visualization of the learned filters.

**Reply:** Confusion matrix is one of the methods to evaluate the model, which has the characteristics of intuitive expression of precision and accuracy. However, to express the same content, at least 8x8 tables are required for the eight-class classification, and the standard evaluation criteria used in this article only need 1x8 tables. There are 56(7 models, 8 data sets, 7x8=56) groups of comparative experiments in this article. If 56 confusion matrix diagrams are provided, it will take up a lot of article space and it is not convenient to compare experimental results. Therefore, this article adopts the standard evaluation criteria: Precision and Accuracy.

The visualization of the learned filters is an auxiliary tool when training the model. Observation for training from scratch can assist in adjusting the parameters of the underlying network to improve the model. This article uses the pre-trained model and model fusion technology. The visualization of the learned filters is of little significance to the model adjustment, so this article does not provide the visualization of the learned filters diagrams.

**About** 5- Poor English”

**Reply:** The original revised manuscript has been repeatedly revised. In this revision, the language has continued to be improved.

**Reply to edit:** In response to the opinion given by the academic editor: "Specifically, the English language should be improved further." We have repeatedly checked the language of the article, corrected the grammatical errors, and unified the translation of professional words. and finally asked a professional thesis translation teacher to review it.

Best regards!

He Qingfang Cheng Guang Ju Huimin

2021-06-08
